# MRI-based analysis of thigh intramuscular fat and its associations with age, sex, and BMI using data from the osteoarthritis initiative data

**DOI:** 10.1038/s41598-024-75005-z

**Published:** 2025-02-20

**Authors:** Gabby B. Joseph, Zehra Akkaya, Wynton M. Sims, Charles E. McCulloch, Michael C. Nevitt, John A. Lynch, Nancy E. Lane, Thomas M. Link

**Affiliations:** 1 Department of Radiology and Biomedical Imaging, University of California, San Francisco, 185 Berry St, Suite 350, San Francisco, CA 94158 USA; 2 Department of Radiology, Faculty of Medicine, Ankara University, Ankara, Turkey; 3 Department of Epidemiology and Biostatistics, University of California, San Francisco, San Francisco, USA; 4 Department of Medicine, University of California, Davis, USA

**Keywords:** Thigh intramuscular fat, MRI, Goutallier grade, Age, Sex, BMI, Risk factors, Medical research

## Abstract

The degree of thigh intramuscular fat in individuals without OA is fundamental for distinguishing natural variations in intramuscular fat from pathological changes. The goals of this study were to estimate the degree of thigh intramuscular fat in individuals without radiographic OA or frequent pain and assess the associations of age, sex, and BMI with the degree of intramuscular fat. Individuals without knee or hip radiographic OA, without total knee/hip arthroplasty, and without frequent knee/hip pain were selected from the OAI database (n = 710). Goutallier Grades (GGs) of the quadriceps and hamstring muscles were assessed based on 3 T MR images on a scale from 0 (normal muscle) to 4 (more fat than muscle). The associations between demographic variables and GG outcomes were evaluated using mixed effects models. The most prevalent GGs among the muscles were Grades 1 and 2; Grade 4 was infrequent (< 1%). Greater BMI (p < 0.001) and age (p < 0.001) were each associated with greater GG. Women had greater GG than men (greatest difference in the vastus medialis: coeff. = 0.214, p < 0.001). At lower BMI, women had greater intramuscular fat than men; at higher BMI, men had greater intramuscular fat than women (p = 0.029 for BMI-sex interaction). While individuals without radiographic OA or frequent pain generally had low thigh intramuscular fat, higher BMI and age were associated with greater intramuscular fat, and GGs were greater in women than men. The relationship between BMI and intramuscular fat was sex-dependent. Thus, demographic variables must be considered when evaluating intramuscular fat.

## Introduction

Obesity is a known risk factor associated with knee osteoarthritis (OA), impacting both mechanical stress and systemic inflammation. Among the various adiposity measures, thigh intramuscular fat (located between and within the muscle fibers) has emerged as an area of interest and is recognized as a potential risk factor for knee OA, which also provides an avenue for treatment^1^.

Research studies on thigh intramuscular fat have predominantly concentrated on the relationship between intramuscular fat and disease burden in knee OA. Studies have reported increased levels of quadriceps intramuscular fat, particularly in the vastus medialis, in individuals with knee OA compared to controls without OA^2,3^. In addition, greater levels of thigh intramuscular fat have been associated with worse cartilage damage^3^, increased clinical symptoms^4^, impairment in neuromuscular activation leading to decreased muscle strength^5^, and decreased physical performance possibly due to an interference with muscle contraction^6^. These studies highlight the significance of intramuscular fat as a potential biomarker and contributor to the progression of knee OA.

However, quantifying thigh intramuscular fat in individuals without OA remains unexplored, leaving a gap in understanding the “normal” levels of intramuscular fat in such populations, which may be associated with sex, age, and body habitus. This knowledge would be fundamental for distinguishing pathological changes from natural variations, consequently enabling informed interpretations of intramuscular fat alterations in the context of knee OA or as a risk factor for knee OA. Semi-quantitative assessment of muscle fat in the thigh muscle can be performed using routine, clinical standard MRI sequences with the universally established Goutallier classification^7^, which does not require sophisticated MRI sequences or post-processing methods and has been studied in the context of OA^8^.

Thus, this study aimed to (1) establish the prevalence of thigh intramuscular fat using the Goutallier classification in individuals without radiographic hip or knee OA and without frequent pain and (2) evaluate the associations between demographic characteristics, including age, sex, and BMI with the degree of intramuscular fat.

## Methods

### Subject selection

This study utilizes data from the Osteoarthritis Initiative (OAI https://www.niams.nih.gov/grants-funding/funded-research/osteoarthritis-initiative)^9^, a multi-center, longitudinal study aimed at assessing biomarkers in knee OA that includes both radiographic and MR imaging. The study protocol, amendments, and informed consent documentation were approved by the institutional review boards of all participating centers.

**Fig. 1:**
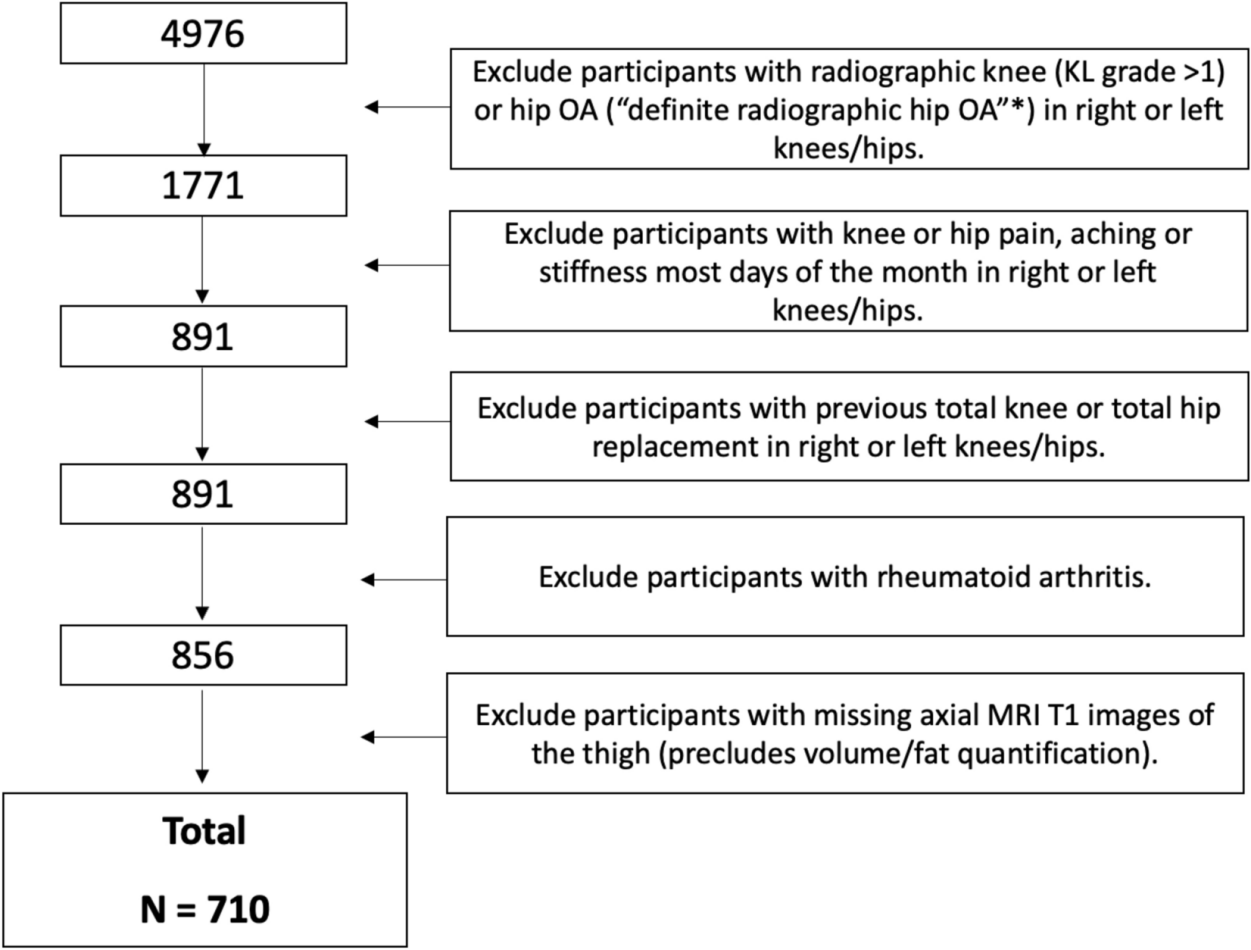
Participant Selection from the OAI database. The ‘*’ designates that hips were classified as “definite RHOA” (modified Croft grade was ≥ 2 and/or grade ≥ 1 femoral or grade ≥ 2 acetabular osteophytes plus definite JSN and/or grade ≥ 2 femoral osteophytes regardless and/or superolateral JSN ≥ 2 or superomedial JSN ≥ 3). Hips were classified as “possible RHOA” when other individual or combinations of indefinite individual radiographic features (IRFs) were present and otherwise considered “normal.” Note that participants with missing data for radiographic hip/knee OA, knee/hip pain, total knee/hip replacement, and rheumatoid arthritis were also excluded. *KL* Kellgren Lawrence.

Participants in this study were selected from the OAI database (n = 4796) at the baseline visit as follows: First, individuals with no radiographic knee OA (Kellgren Lawrence grades (KL) 0 and 1) and no definite hip radiographic OA (described below) in right or left knees or hips were selected (n = 1771). Of those, individuals with knee or hip pain, aching, or stiffness (right or left sides) on most days of the month were excluded (n = 891), and individuals with previous total knee or hip arthroplasty (n = 891) were also excluded. Additionally, those with rheumatoid arthritis were excluded (n = 856). Of the 856 individuals, 710 had axial MRI thigh images available for analysis (which were used to assess intramuscular fat). Thus, overall, 710 participants were included in the analysis of this study, as shown in Fig. 1.

## Imaging

### Radiographs

Standardized bilateral standing posterior-anterior fixed flexion knee radiographs were acquired in all subjects in the OAI. Baseline KL grades^10^, which were measured in the right and left knees from fixed flexion knee radiographs, were provided in the OAI database. Individuals without radiographic knee OA (KL grades 0 or 1) were included in this study. Weight-bearing pelvis radiographs at baseline were also acquired in the OAI. Pelvis radiographs were assessed for hip OA using the OARSI atlas as previously described^11^. Individuals with hips (right or left) previously graded as definite radiographic OA were excluded from this study^12,13^.

### Magnetic resonance (MR) imaging and image analysis

MR images were obtained using four identical 3.0 Tesla (Siemens Magnetom Trio, Erlangen, Germany) scanners in Columbus, Ohio; Baltimore, Maryland; Pittsburgh, Pennsylvania; Pawtucket, Rhode Island. Muscle and fat volume measurements were performed using an axial thigh T1 weighted spin echo sequence (T1w SE) [repetition time (TR)/echo time (TE); spatial resolution; field of view (FOV); slice thickness; gap] [500 ms/10 ms; 0.977 mm × 0.977 mm; 500 mm; 5 mm; 0 mm] at baseline. Acquisition of the axial T1W MRI images of bilateral thighs was performed in a standardized location (starting 10 cm proximal to the distal epiphysis of the femur and extended 7.5 cm proximally; covering approximately the middle 33% of the femoral length). Further details about the image acquisition are available in the OAI MR protocol^14^.

Intramuscular fat was analyzed using the semi-quantitative Goutallier Grading (GG) scale^7^. This five-level grading system analyzes the degree of muscle fat infiltration where grade 0 is normal muscle without fat infiltration, grade 1 demonstrates some fatty streaks, grade 2 shows less fat than muscle, grade 3 has equal amounts of fat and muscle, and grade 4 more fat than muscle is shown. The following muscles were graded (right and left thighs): knee extensors [quadriceps, including the vastus medialis (VM), vastus lateralis (VL), vastus intermedius (VI) and the rectus femoris (RF) muscles] as well as the knee flexors [hamstrings, including the semimembranosus (SM), semitendinosus (ST), and biceps femoris (BF) muscles]. All images were analyzed by 2 trained observers.

Intra- and inter-reader reproducibility of the Goutallier gradings were obtained from the readings of the same trained observers. Each observer independently graded each muscle in 100 cases (200 thighs) for inter-reader reproducibility. A subsequent independent review of the same 100 cases (200 thighs) by one observer was used to quantify intra-reader reproducibility. This systematic approach provided insights into the consistency of GG within and between readers, enhancing the overall reliability of this study’s findings.

### Knee and hip pain

Right and left knee and hip pain were assessed at baseline. Individuals were asked whether they had pain, aching or stiffness in the hip or knee on most days of a month during the past year^15^. Those who said ‘yes’ were considered to have *frequent knee or hip pain*. Individuals with frequent knee or hip pain at baseline were excluded from this study.

### Physical activity scale for the elderly

The Physical Activity Scale for the Elderly (PASE)^16^ was utilized to assess the levels of physical activity among participants. The PASE score, encompassing daily activities, leisure pursuits, and occupational engagements, provided a comprehensive measure to gauge the participants’ overall physical activity levels.

### Statistical analysis

Statistical analysis was performed using STATA version 18 software (StataCorp LP, College Station, TX). Inter- and Intra-reader reproducibility was assessed by calculating weighted Cohen’s Kappa and using cluster-resampled bootstrapping with 1000 repetitions to derive a 95% CI while accounting for clustered observations (two thighs per person). GG grades were tabulated, and percentages of each grade in each quadriceps and hamstring muscle type were quantified. Mixed effects models were used to separately assess the relationship of each demographic variable (age, sex, and BMI) and muscle type with GG outcomes by including an interaction between each demographic characteristic and muscle type^17^. To enhance the interpretability of the mixed-effects model (specifically differences in GGs between muscle groups), centering techniques were applied to key predictor variables. The variable ‘BMI’ was centered by subtracting each individual’s BMI value from the mean BMI, while the variable ‘age’ was centered by subtracting each individual’s age from the mean age. Models accounted for two thighs per person and seven muscle types per thigh and were adjusted for BMI, age, sex, and Physical Activity Scale for the Elderly (PASE).

In the mixed models, if the interaction between each demographic characteristic and muscle type was statistically significant, individual models in each muscle type were implemented. Coefficients for continuous predictors (BMI, age) represent change in outcome (GG) per one unit change in the predictor. The coefficient for sex represents the difference in GG between males and females (males are the reference group).

In addition, to evaluate potential variations in the relationship between BMI and GG based on sex, a BMI-sex-muscle type interaction, along with corresponding two-way interactions (BMI-sex, BMI-muscle type, sex-muscle type) were added to the initial model described above. Starting with the highest order interaction, the interaction terms were removed from the model if they were not statistically significant. A p-value of < 0.05 was considered statistically significant. Model assumptions were checked to ensure the validity of the mixed models used in the analysis. Additionally, checks for linearity were performed by including quadratic and higher order terms for predictors, and the relationships were confirmed to be adequately linear.

## Results

### Participant characteristics

Participants (n = 710) had a mean (± SD) age of 59.8 ± 9.0 years, a mean (± SD) BMI of 27.1 ± 4.3 kg/m^2^ and 55.5% of subjects were female (n = 394). KL grades for knees showed 73.6% of knees with a grade of 0, while 26.4% of knees had a KL grade of 1. While all participants with pain, aching, or stiffness in the hip or knee on most days of a month during the past year were excluded, pain assessment using the Western Ontario and McMaster Universities Arthritis Index (WOMAC) Pain Score [range 0–20] revealed minimal pain with median [IQR] scores of 0 ^1^ for the right and left knees. The remaining participant characteristics are listed in Table 1.

**Table 1:** Participant characteristics.

Participant characteristics	Summary
N	710
Age (years)	59.8 (9.0)
Sex	
Males	316 (44.5%)
Females	394 (55.5%)
BMI (kg/m^2^)	27.1 (4.3)
KL grade, right knee	
0	523 (73.6%)
1	187 (26.4%)
KL grade, left knee	
0	523 (73.6%)
1	187 (26.4%)
WOMAC Pain Score, right knee*	0 [1]
WOMAC Pain Score, left knee*	0 [1]
RHOA, right hip	
None	639 (90.0%)
Possible	71 (10.0%)
RHOA, left hip	
None	642 (90.4%)
Possible	68 (9.6%)
Flexion max force, right knee [N]	152.5 (69.6)
Flexion max force, left knee [N]	151.3 (71.3)
Extension max force, right knee [N]	378.4 (131.8)
Extension max force, left knee [N]	355.0 (127.9)

Continuous Variables: Mean (Standard deviation); Categorical Variables: Frequency (Percent %). *OA* Osteoarthritis, *WOMAC* Western Ontario and McMaster Universities Arthritis Index, *KL* Kellgren Lawrence, *RHOA* Radiographic Hip OA.

*Note that median [IQR] are listed for WOMAC pain scores.

### Reproductivity

Table 2 lists weighted kappa statistics to evaluate both inter-observer and intra-observer reproducibility for various muscle measurements, with kappa values indicating the degree of agreement (0 representing no agreement and 1 indicating perfect agreement). For *inter-observer* reproducibility, kappa values indicate a mostly high degree of agreement, ranging from kappa = 0.67 (bootstrapped 95% CI 0.54–0.80) for the rectus femoris (moderate agreement) to kappa = 0.86 (bootstrapped 95% CI 0.78–0.95) for the biceps femoris (strong agreement)^18^. *Intra-observer* reproducibility demonstrates even greater consistency, with kappa values ranging from kappa = 0.85 (bootstrapped 95% CI 0.79–0.91) for the biceps femoris to kappa = 0.92 (bootstrapped 95% CI 0.88–0.97) for the semitendinosus. These findings highlight the robust reliability of the measurements both within and between observers for all the muscles examined.

**Table 2:** Inter- and Intra-observer reproducibility in each muscle type. Inter- and Intra-reader reproducibility was assessed by calculating weighted Cohen’s Kappa and using cluster-resampled bootstrapping with 1000 repetitions to derive a 95% CI while accounting for clustered observations (two thighs per person).

Muscle	Kappa	Bootstrap standard error	Bootstrap 95% confidence interval
Inter-observer reproducibility			
Vastus Medialis	0.82	0.06	0.70–0.93
Vastus Lateralis	0.73	0.06	0.61–0.86
Vastus Intermedius	0.80	0.06	0.68–0.92
Rectus Femoris	0.67	0.07	0.54–0.80
Semimembranosus	0.68	0.05	0.58–0.78
Semitendinosus	0.70	0.06	0.57–0.83
Biceps Femoris	0.86	0.04	0.78–0.95
Intra-observer reproducibility			
Vastus Medialis	0.88	0.03	0.82–0.95
Vastus Lateralis	0.88	0.03	0.82–0.94
Vastus Intermedius	0.91	0.03	0.85–0.96
Rectus Femoris	0.89	0.02	0.83–0.95
Semimembranosus	0.86	0.02	0.81–0.92
Semitendinosus	0.92	0.02	0.88–0.97
Biceps Femoris	0.85	0.03	0.79–0.91

### Prevalence of Goutallier grades

**Fig. 2:**
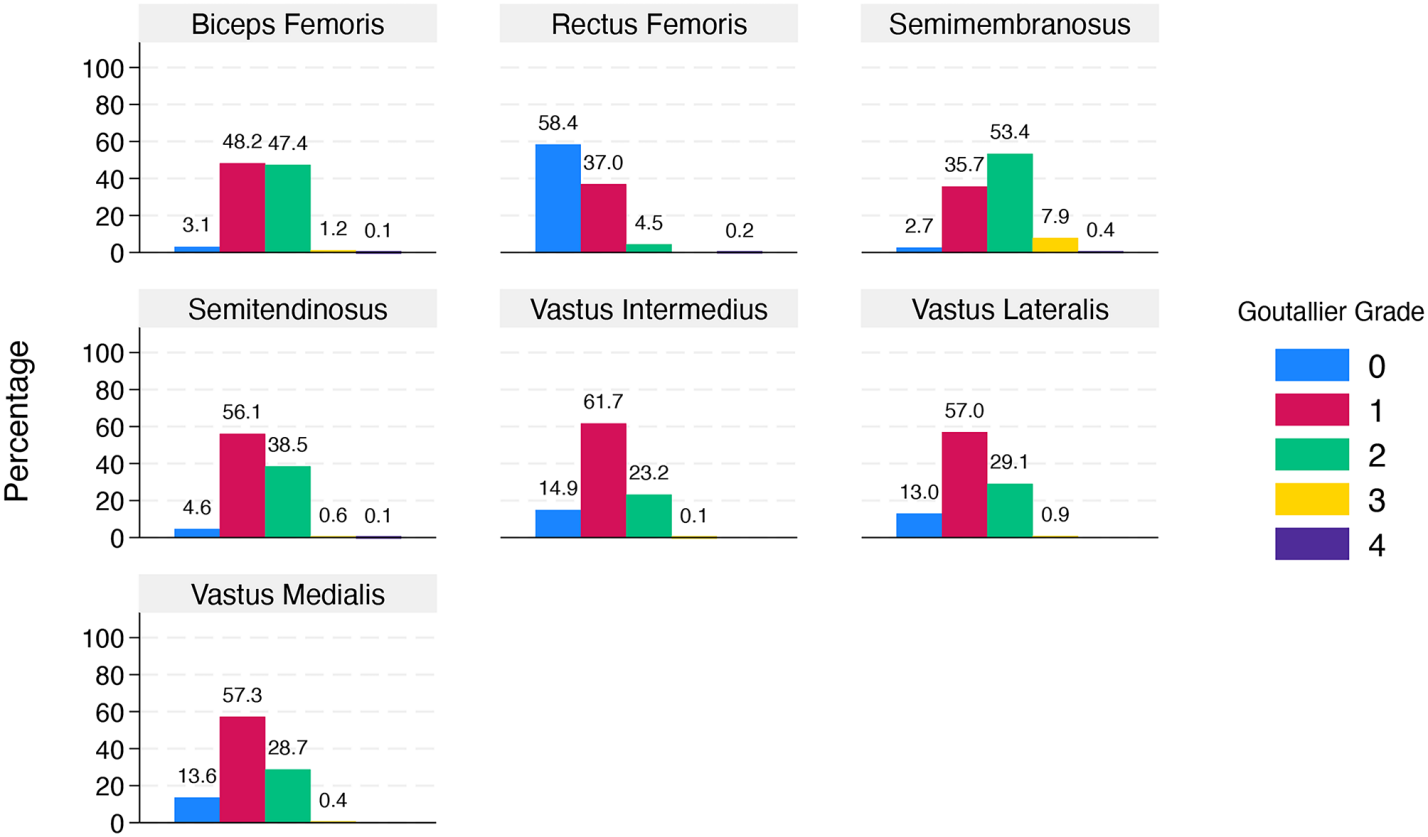
Distribution of goutallier grades among various muscles. The grades range from 0 to 4, with different colors representing each grade.

The most prevalent GGs among the muscles were Grades 1 and 2, with the vastus intermedius muscle having the highest Grade 1 prevalence (61.7%) and the semimembranosus muscle having the highest Grade 2 prevalence (53.4%). Among all muscles except the rectus femoris muscle, the range for Grade 0 varied between 2.7 and 14.9%, with the rectus femoris muscle notably displaying the highest occurrence of Grade 0 at 58.4%. Conversely, Grade 4 was infrequent across the muscles, ranging from 0.1% (semitendinosus and biceps femoris) to 0.4% (semimembranosus) (Fig. 2).

### Interactions between demographics and muscle type

**Fig. 3:**
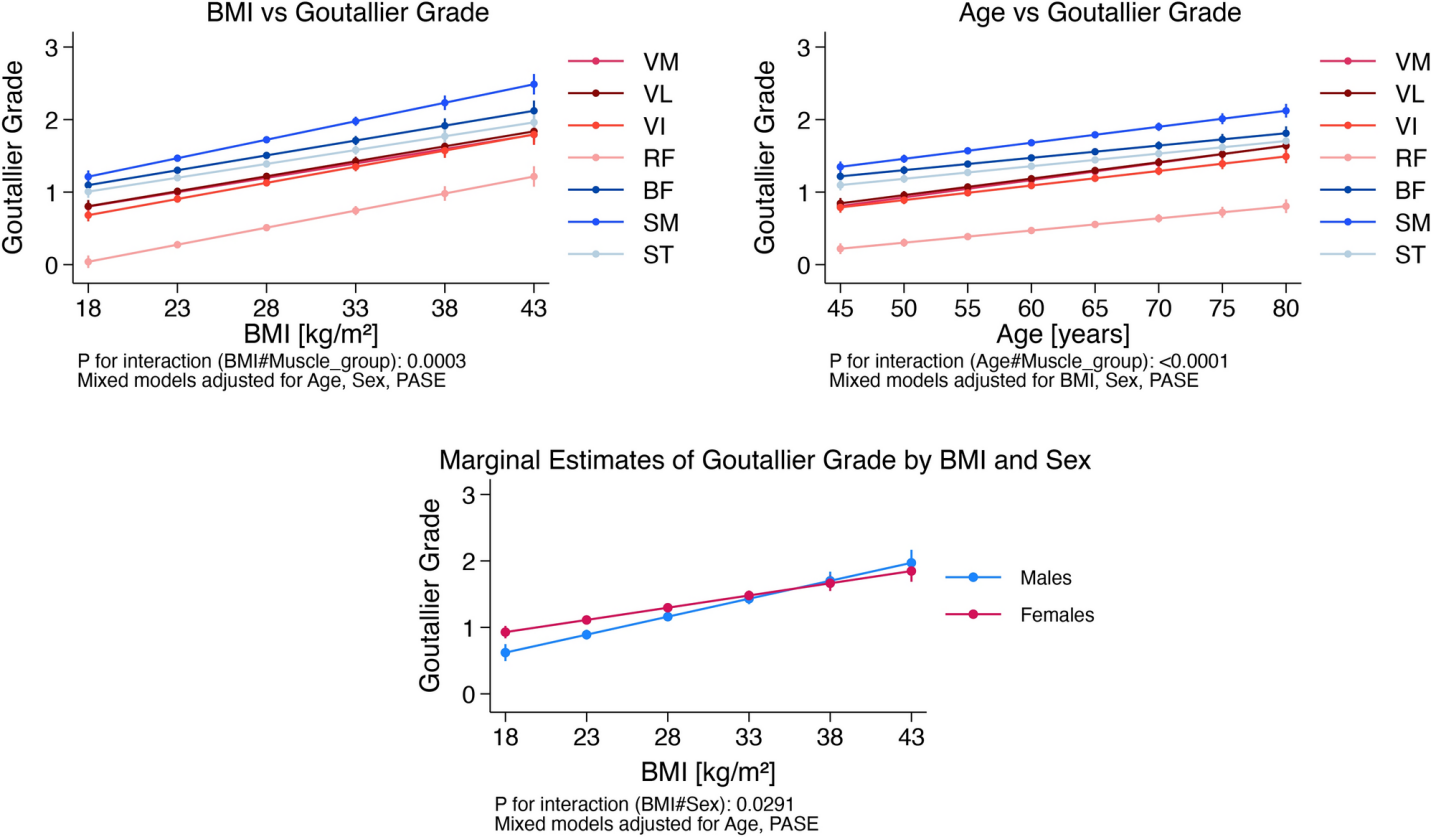
The graphs illustrate the associations between BMI and age with GG. In the graphs on the top row, the red color family designates the quadriceps, and the blue color family designates the hamstrings. The graph (bottom row) demonstrates statistically significant interactions between BMI and sex on GG grade (p = 0.029), suggesting that the effects of BMI on GG grade vary by sex. Error bars represent 95% CIs.

The interactions between muscle type and BMI (p = 0.0003), age (p < 0.0001), and sex (p < 0.0001) were statistically significant, suggesting that the associations between demographics and GG vary by muscle type (Fig. 3).

Based on centered values for BMI (average 27.1 kg/m^2^) and age (average 59.8 years), the rectus femoris had the lowest GG (adjusted mean = 0.47, 95% CI [0.43, 0.50]), while the semimembranosus had the highest (adjusted mean = 1.68, 95% CI [1.64, 1.71]). Relative to rectus femoris, significantly greater GGs were observed in the vastus medialis at 0.69 (95% CI [0.67, 0.72], p < 0.0001), vastus lateralis at 0.71 (95% CI [0.69, 0.74], p < 0.0001), vastus intermedius at 0.62 (95% CI [0.59, 0.65], p < 0.0001), biceps femoris at 1.00 (95% CI [0.98, 1.03], p < 0.0001), and semitendinosus at 0.89 (95% CI [0.86, 0.91], p < 0.0001), highlighting the variability in GGs across muscle types.

### Associations between demographics and muscle type

**Fig. 4:**
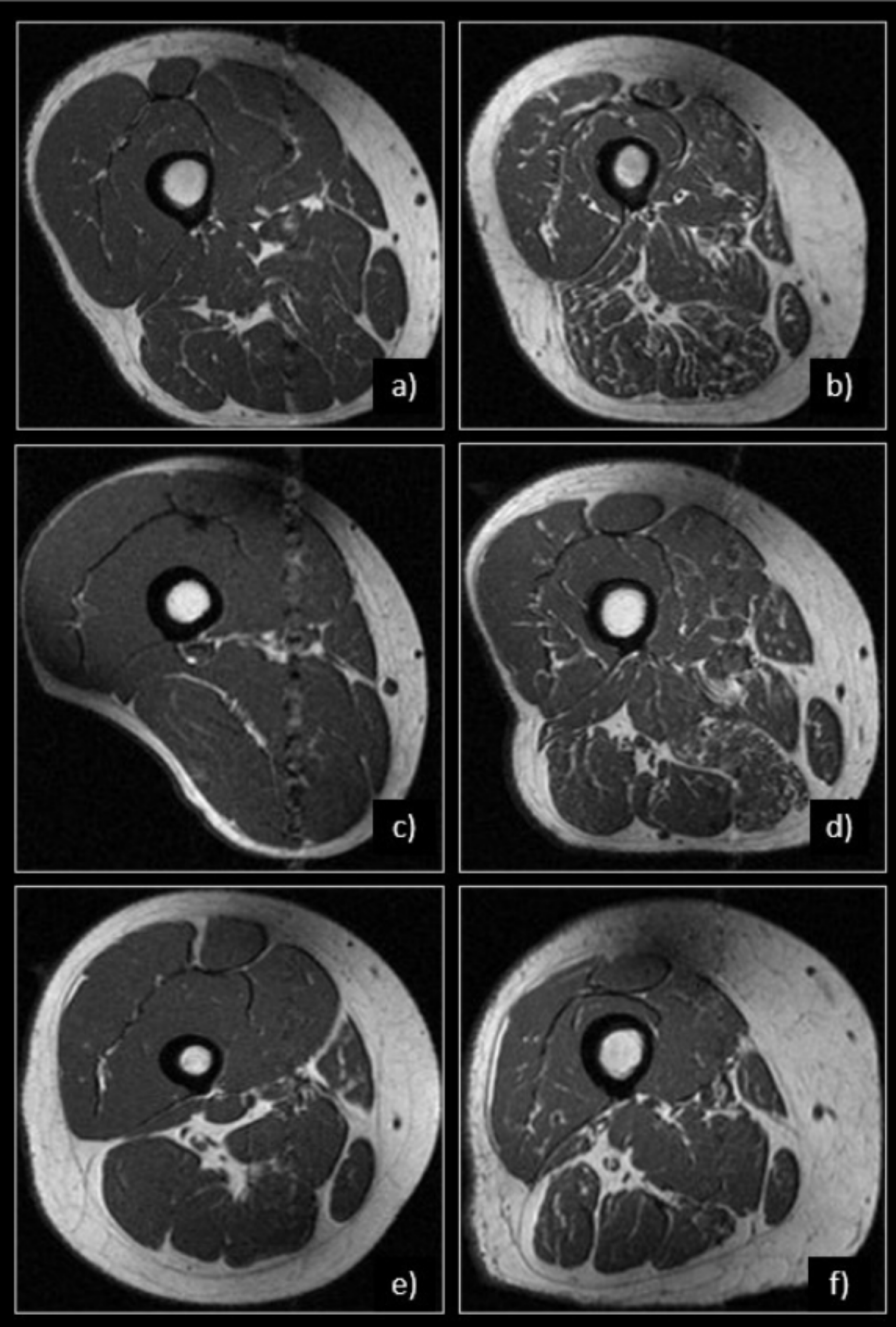
Row 1: men vs. women. Representative MR images of right thighs from 65-year-old man (**a**) and a 61-year-old woman (**b**), both with a of BMI = 34 kg/m^2^ are shown. Note the abundance of fatty streaks in all muscle groups, particularly in the hamstrings in (**b**) compared to (**a**). GGs in (**b**) ranged from 2–3 for all muscles, while GGs in (**a**) ranged from 0–1 for all muscles. Row 2: normal weight vs. obese. Representative MR images of right thighs from a 58-year-old man with BMI = 19 kg/m^2^ (**c**) and 60-year-old man with BMI = 36 kg/m^2^ (**d**) are shown. Despite similar ages in both men, higher GGs, ranging between 1 and 3, were recorded in the participant with higher BMI (**d**) compared to the lean participant (**c**) with GGs ranging between 0 and 1. Row 3: younger vs. older. Representative MR Images of right thighs from a 48-year-old woman with BMI = 32 kg/m^2^ (**e**) and a 71-year-old woman with BMI = 31 kg/m^2^ (**f**) are shown. Note the higher abundance of fatty streaks within all muscle compartments in (**f**), representing increased intramuscular fat with older age. GGs ranged between 0–1 in (**e**) and 0–3 in (**f**).

Since the interaction between each demographic variable (BMI, age, and sex) and muscle type for the association with GGs was statistically significant, individual models for each muscle type were applied (Table 3). A positive relationship between BMI and GG was evident (p < 0.001 for all muscles), with coefficients (representing change in GG for every 1 unit increase in BMI) ranging from 0.038 in the semitendinosus to 0.051 in the semimembranosus. Similarly, a positive association (p < 0.001) was observed between age and GG (coefficients ranging from 0.017 in the semitendinosus and biceps femoris to 0.023 in the vastus medialis and vastus lateralis). Women had greater GG than men in all muscles (greatest difference in the vastus medialis: coeff. = 0.214, p < 0.001, and smallest difference in the rectus femoris (coeff. = 0.088, p = 0.037) and semimembranosus (coeff. = 0.088, p = 0.049)), Fig. 4.

**Table 3:** Associations between demographics and GG grades. Mixed models accounted for two thighs per person and were adjusted for BMI, age, sex, and Physical Activity Scale for the Elderly (PASE). Coefficients for continuous predictors (BMI, age) represent change in outcome (GG) per one unit change in the predictor. The coefficient for sex represents the difference in GG between males and females (males are the reference group).

	BMI (kg/m^2^)	Age (years)	Sex (males are reference)
**Quadriceps**			
Coefficient	0.043	0.022	0.164
95% CI	0.033 0.053	0.017 0.027	0.074 0.253
p-value	< 0.001	< 0.001	< 0.001
Vastus Medialis			
Coefficient	0.041	0.023	0.214
95% CI	0.031 0.051	0.018 0.028	0.128 0.300
p-value	< 0.001	< 0.001	< 0.001
Vastus Lateralis			
Coefficient	0.042	0.023	0.177
95% CI	0.032 0.052	0.018 0.028	0.090 0.264
p-value	< 0.001	< 0.001	< 0.001
Vastus Intermedius			
Coefficient	0.045	0.020	0.168
95% CI	0.035 0.054	0.015 0.025	0.085 0.251
p-value	< 0.001	< 0.001	< 0.001
Rectus Femoris			
Coefficient	0.046	0.018	0.088
95% CI	0.037 0.055	0.014 0.023	0.005 0.170
p-value	< 0.001	< 0.001	0.037
**Hamstrings**			
Coefficient	0.049	0.020	0.075
95% CI	0.040 0.059	0.015 0.025	-0.011 0.160
p-value	< 0.001	< 0.001	0.089
Biceps Femoris			
Coefficient	0.040	0.017	0.130
95% CI	0.031 0.049	0.012 0.021	0.052 0.209
p-value	< 0.001	< 0.001	0.001
Semimembranosus			
Coefficient	0.051	0.022	0.088
95% CI	0.041 0.061	0.017 0.027	0.000 0.176
p-value	< 0.001	< 0.001	0.049
Semitendinosus			
Coefficient	0.038	0.017	0.172
95% CI	0.029 0.047	0.012 0.022	0.093 0.252
p-value	< 0.001	< 0.001	< 0.001

### BMI–sex interactions and muscle type

The interaction between BMI-sex [but not the interactions of age-sex (p = 0.69) or BMI-age (p = 0.41)] was statistically significant (p = 0.029), demonstrating that the association between BMI and GG varied by sex and was similar for all muscles (BMI-sex-muscle type interaction was not statistically significant, p = 0.21). At lower BMI, women had greater intramuscular fat than men, but at higher BMI, men had greater intramuscular fat than women (Fig. 3).

## Discussion

This study suggests “reference” values for intramuscular fat (assessed by GG) in individuals without frequent pain and without radiographic knee or hip OA. These individuals generally exhibited low GGs of thigh intramuscular fat (primarily grades 1 and 2, varying by muscle type). Greater BMI and older age were each associated with higher levels of intramuscular fat, and women tended to have higher levels of intramuscular fat than men. The relationship between BMI and intramuscular fat varied by sex, possibly due to sex-specific differences related to hormonal changes in obesity. The “reference” values presented in this manuscript represent typical intramuscular fat levels in individuals without frequent knee or hip pain and without radiographic evidence of OA. They can be used as benchmarks to assess deviations in individuals with joint degeneration or other musculoskeletal conditions.

Previous studies utilizing MRI to assess thigh intramuscular fat have reported positive associations with age^3^ and BMI^19^. The present study aligns with these findings but has a larger sample size (n = 710 vs previous studies: n = 69 and n = 63, respectively) and exclusively assesses individuals without OA. Age-related accumulation of intramuscular fat may be due to factors including hormonal changes, decreased physical activity (contributing to muscle mass reduction and increased fat storage within muscle tissue)^20^, and alterations in muscle metabolism^21^. One potential mechanism described by Li et al.^22^, suggests that aging accompanies a redistribution in muscle and adipose mass due to an imbalance between protein synthesis and proteolysis, leading to a decline in skeletal muscle mass, strength, and function (sarcopenia). Sarcopenia is also associated with muscle fiber hypertrophy (reduced diameter of type II glycolytic myofibers), which leads to fatty infiltration in the muscle fibers. In parallel, physiological changes during obesity can contribute to increases in intramuscular fat. Obesity can trigger muscle progenitor cells to transform into adipocyte‐like cells due to inflammatory signals, which leads to decreases in muscle regeneration and increases in intramuscular fat. This process contributes to a cycle in which intramuscular fat accumulation impairs muscle function and contributes to the progression of sarcopenic obesity. The presence of intramuscular fat also exacerbates insulin resistance and inflammation, further hindering muscle repair and reinforcing the cycle of muscle loss and obesity^22^. Overall, a multitude of interrelated factors may contribute to increased intramuscular fat with aging and obesity, including hormonal changes, alterations in lipid metabolism, reduced physical activity, and inflammation.

In this study, the associations between BMI and intramuscular fat varied by sex. At lower BMI values, women had greater intramuscular fat than men; however, obese men had greater intramuscular fat than obese women. These results may be potentially associated with decreases in testosterone levels associated with obesity in men^23^, which may lead to greater visceral and intramuscular fat deposition. In addition, obese men have “elevated glucose levels and lower adiponectin levels promoting intraabdominal adiposity and insulin resistance^24^” compared to obese women, a finding which may also explain the results observed in this study.

Of all the muscles examined in this study, the rectus femoris muscle has the least intramuscular fat (as previously reported^21^) potentially due to its unique characteristics. The rectus femoris’ fibers are smaller and denser, and it is the only bipartite muscle in the quadriceps that both contributes to motor control and is influenced by the hip joint^25^. In addition, physical activity can engage various muscles differently based on the activity type^26^, potentially influencing the variation in fat infiltration among muscle groups.

Establishing “reference” levels of intramuscular fat in individuals without OA provides a comparative reference against which elevated levels of intramuscular fat observed in knee OA patients can be assessed. This knowledge is fundamental for distinguishing pathological changes from natural variations, enabling a more nuanced interpretation of intramuscular fat alterations in the context of knee OA. Additionally, insights into “normative” levels of intramuscular fat can inform preventive strategies, potentially identifying thresholds that, when surpassed, may indicate an increased risk of developing knee OA. Such preventive strategies may include resistance training 3 days/week in adults, which has been reported to be associated with decreased thigh intramuscular adipose tissue^27^, and brisk walking which was preventive of fatty infiltration of muscle in older individuals (randomized control trial)^28^. Overall, this study may serve as a reference database for intramuscular fat in individuals without radiographic OA or pain, with implications for advancing strategies for early intervention and personalized care in individuals at risk for knee OA.

Although physical activity was not a primary focus of this study, we performed a sub-analysis to explore its relationship with intramuscular fat, (adjusting for age, sex, and BMI). This analysis showed that higher PASE scores were significantly associated with lower intramuscular fat in the hamstrings, with no significant associations observed in the quadriceps. Further research is warranted to understand the interplay between physical activity and muscle fat composition.

Several limitations are pertinent to this study. The GG scoring system is semi-quantitative in nature; a more precise evaluation would entail quantitative measurements of the intra-muscular fat fraction. However, the advantage of the GG is that it does not require advanced MRI techniques and post-processing algorithms, which may also have reproducibility issues and are not comparable across different MRI scanners, particularly those from different manufacturers. In addition, while it would be ideal for the enrolled individuals to be asymptomatic, our subject cohort excluded individuals with pain most days of the month in the knee and hip. Individuals in this study had very low levels of pain (1.0 ± 1.7 WOMAC pain in the right knee and 0.8 ± 1.6 in the left knee out of a range 0–20, with a median score of 0 in both knees), which were similar to the mean pain scores in the OAI database overall. Another potential limitation is that the specific selection criteria, excluding individuals with frequent hip or knee pain and radiographic OA, may limit the generalizability of the findings to populations with varying levels of joint health. While the participants included in this study did not have frequent knee or hip pain or radiographic evidence of knee or hip OA, they were recruited based on OAI inclusion criteria^29^ that included risk factors for OA, such as being overweight (determined by gender- and age-specific cut-points) or a history of knee injury. As a result, this cohort may not represent a true reference group, and this limitation should be considered when interpreting the study’s findings. Overall, despite these limitations, we believe that our study provides an important contribution to the field, as it establishes the first large dataset of “reference” intramuscular fat GG grades in individuals without radiographic OA and frequent pain.

## Conclusions

In this study, older age and greater BMI were associated with greater levels of thigh intramuscular fat in individuals without radiographic hip or knee OA and without frequent pain. The relationship between BMI and intramuscular fat varied by sex, potentially due to sex-specific hormonal changes that occur with obesity. These findings underscore the complexity and interplay between biological aging, adiposity, and sex-specific changes in thigh intramuscular fat accumulation, which may eventually help further knowledge of muscle quality changes in relation to OA and develop preventative strategies.

## Data Availability

The datasets generated and/or analyzed during the current study are available in the Osteoarthritis Initiative (OAI) repository, [https://nda.nih.gov/oai].

## Abbreviations

BF: Biceps femoris
BMI: Body mass index
TE: Echo time
FOV: Field of view
GG: Goutallier grading
KL: Kellgren Lawrence grades
MRI: Magnetic resonance imaging
OA: Osteoarthritis
OAI: Osteoarthritis initiative
PASE: Physical activity scale for the elderly
RF: Rectus femoris
TR: Repetition time
SM: Semimembranosus
ST: Semitendinosus
T1W: T1 weighted
VI: Vastus intermedius
VL: Vastus lateralis
VM: Vastus medialis
WOMAC: Western Ontario and McMaster Universities Arthritis Index
